# Modern approaches to psychopharmacotherapy of anorexia nervosa and bulimia nervosa

**DOI:** 10.1192/j.eurpsy.2021.952

**Published:** 2021-08-13

**Authors:** I. Belokrylov, E. Okonishnikova, T. Lineva

**Affiliations:** 1 Department Of Psychiatry And Medical Psychology, Peoples Friendship University of Russia (RUDN University), Moscow, Russian Federation; 2 Department Of Psychiatry And Medical Psychology, RUDN University Moscow., Moscow, Russian Federation; 3 Department Of Psychiatry And Medical Psychologi, Peoples Friendship University of Russia (RUDN University), Moscow, Russian Federation

## Abstract

**Introduction:**

Currently, there are no ideal medications for treating anorexia nervosa (AN) and bulimia nervosa (BN). This is due to the variety of symptoms from the mental and somatic spheres.

**Objectives:**

Describe the modern methods of psychopharmacotherapy AN and BN.

**Methods:**

Data from available publications on the topic of psychopharmacotherapy AN and BN, and long-term practical experience of research staff the Department of psychiatry and medical psychology RUDN University, Moscow.

**Results:**

Therapy includes antidepressants (AD) - serotonin reuptake inhibitors (SSRIs), antipsychotics and tranquilizers. AD groups of SSRIs reduce most of the symptoms AN and BN - depressive disorders, anxiety, obsessive and compulsive symptoms, episodes of overeating and purifying behavior, suicidal thoughts, and reduce the frequency of relapses. With severe and persistent dysmorphophobia, a high degree of impulsivity, and psychopathic behavior second-generation antipsychotics Quetiapine, Olanzapine, Risperidone and Aripiprazole are used. Benzodiazepine tranquilizers (Lorazepam) are used in small doses and as additional therapy. Data from the European national guidelines for the treatment of AN and BN very different, and the world Federation of societies for biological psychiatry (WFSBP) does not provide specific recommendations at all. There are many reasons for disagreement and lack of specificity regarding drug selection, including the lack of an equally solid evidence base, that reflects the modern state of research on the psychopharmacological treatment of eating disorders.

**Conclusions:**

In General, therapy AN and BN should be comprehensive - psychopharmacotherapy, psychotherapy, diet therapy, social rehabilitation. Treatment should be carried out both in the hospital and on outpatient basis and should be decided individually.

**Conflict of interest:**

No significant relationships.

